# Participation of heparin binding proteins from the surface of *Leishmania (Viannia) braziliensis* promastigotes in the adhesion of parasites to *Lutzomyia longipalpis* cells (Lulo) *in vitro*

**DOI:** 10.1186/1756-3305-5-142

**Published:** 2012-07-17

**Authors:** Luzia Monteiro de Castro Côrtes, Mirian Claudia de Souza Pereira, Franklin Souza da Silva, Bernardo Acácio Santini Pereira, Francisco Odêncio de Oliveira Junior, Renata Oliveira de Araújo Soares, Reginaldo Peçanha Brazil, Leny Toma, Carolina Meloni Vicente, Helena Bonciani Nader, Maria de Fátima Madeira, Felio J Bello, Carlos Roberto Alves

**Affiliations:** 1 Laboratório de Biologia Molecular e Doenças Endêmicas, Av. Brasil 4365, Rio de Janeiro - CEP, 21040-360, Brazil; 2 Laboratório de Ultraestrutura Celular, Av. Brasil 4365, Rio de Janeiro - CEP, 21040-360, Brazil; 3 Laboratório de Bioquímica e Fisiologia de Insetos - IOC - Fiocruz, Av. Brasil 4365, Rio de Janeiro - CEP, 21040-360, Brazil; 4 Laboratório de Vigilância em Leishmanioses - IPEC - Fiocruz, Av. Brasil 4365, Rio de Janeiro - CEP, 21040-360, Brazil; 5 Departamento de Bioquímica Universidade Federal de São Paulo, UNIFESP, SP, Brazil; 6 Universidad Del Rosario, Escuela de Medicina, Carrera 24 no 63 C-69, Bogotá, D.C, Colombia

**Keywords:** *L. (V.) braziliensis*, Promastigotes, Lulo cells, Glycosaminoglycans, Surface plasmon resonance

## Abstract

**Background:**

*Leishmania (V.) braziliensis* is a causative agent of cutaneous leishmaniasis in Brazil. During the parasite life cycle, the promastigotes adhere to the gut of sandflies, to avoid being eliminated with the dejection. The Lulo cell line, derived from *Lutzomyia longipalpis* (Diptera: Psychodidae), is a suitable *in vitro* study model to understand the features of parasite adhesion*.* Here, we analyze the role of glycosaminoglycans (GAGs) from Lulo cells and proteins from the parasites in this event.

**Methods:**

Flagellar (F_*f*_) and membrane (M_*f*_) fractions from promastigotes were obtained by differential centrifugation and the purity of fractions confirmed by western blot assays, using specific antibodies for cellular compartments. Heparin-binding proteins (HBP) were isolated from both fractions using a HiTrap-Heparin column. In addition, binding of promastigotes to Lulo cells or to a heparin-coated surface was assessed by inhibition assays or surface plasmon resonance (SPR) analysis.

**Results:**

The success of promastigotes subcellular fractionation led to the obtainment of F_f_ and M_f_ proteins, both of which presented two main protein bands (65.0 and 55.0kDa) with affinity to heparin. The contribution of HBPs in the adherence of promastigotes to Lulo cells was assessed through competition assays, using HS or the purified HBPs fractions. All tested samples presented a measurable inhibition rate when compared to control adhesion rate (17 ± 2.0% of culture cells with adhered parasites): 30% (for HS 20μg/ml) and 16% (for HS 10μg/ml); HBP M_f_ (35.2% for 10μg/ml and 25.4% for 20μg/ml) and HBP F_f_ (10.0% for 10μg/ml and 31.4% for 20μg/ml). Additionally, to verify the presence of sulfated GAGs in Lulo cells surface and intracellular compartment, metabolic labeling with radioactive sulfate was performed, indicating the presence of an HS and chondroitin sulfate in both cell sections. The SPR analysis performed further confirmed the presence of GAGs ligands on *L. (V.) braziliensis* promastigote surfaces.

**Conclusions:**

The data presented here point to evidences that HBPs present on the surface of *L. (V.) braziliensis* promastigotes participate in adhesion of these parasites to Lulo cells through HS participation.

## Background

The leishmaniases are vector-borne anthropozoonotic diseases, which are caused by parasites of the genus *Leishmania*. These parasites are inoculated into their mammal hosts during the blood meal of the phlebotomine sandflies, which are widely spread in tropical and subtropical regions (http://www.who.int/leishmaniasis/en/). When *Leishmania* parasites infect human hosts, they can induce an array of clinical manifestations, varying from tegumentary (mucocutaneous, cutaneous and diffuse) to visceral infections. In Brazil, *Leishmania (Viannia) braziliensis* is the main species related to the cutaneous and mucocutaneous forms of the disease [1], and its primary vectors are *Lutzomyia (Nyssomyia) intermedia* and *Lutzomyia (Nyssomyia) whitmani*[2]. The difficulty of controlling the disease in endemic areas is a consequence of the absence of effective vaccines or drug therapies against the different species of *Leishmania,* a consequence of the complexity of host-pathogen interactions [3].

During their life cycle, these parasites undergo distinct morphological stages, as promastigotes in the sandfly host and amastigotes in the mammalian host, each one adapted to its microenvironment [4]. Such adaptations are pivotal for a successful completion of the parasite life cycle. For example, the development of promastigotes, coming from a contaminated blood meal, to the procyclical form in the peritrophic matrix formed in the gut of the sandfly host after feeding [5], may be related to parasites escaping from this matrix and adhering to the microvilli of epithelial cells in the stomach [6]. This is an example of an essential step in maintainance of the cycle and may be a factor in the selection of infective and non-infective strains [5]. The promastigotes living within the sandfly gut, present a flagellum that is responsible for their motility and, also, plays a role in the attachment to sandfly gut [7]. Although several organic components of *Leishmania* spp have been the subject of many studies for understanding the biological cycle of these parasites in the mammalian, studies about the role of such components in the interaction with the insect vector are less abundant. Parasite surface components that have been shown to act in parasite-host interaction include glycoconjugates from promatigotes, as the glycosylated major surface protein of 63 kDa (gp63) [8], lipophosphoglycan (LPG) or proteophosphoglycan (PPG) [9]. It has been described that the down-regulation of gp63 in a *Leishmania (Leishmania) amazonensis* clone adversely affects its development in the neotropical sand fly, *Lutzomyia longipalpis*. A possible role for LPG in the parasite interaction with the intestinal epithelium of sandfly has been proposed [10-12], where this molecule would prevent the promastigotes to be excreted with the dejection, while PPG in filamentous form (fPPG) seems to play a mechanical role, blocking the foregut and, thus, inducing regurgitation, which is advantageous for parasite infection of mammal host [13].

The state of the art on *Leishmania* spp life cycle studies includes a recent discussion on the use of insect cell lines to understand the fine interactions that occur between these parasites and their invertebrate hosts. For example, Lulo cells, a cell lineage derived from *Lutzomyia longipalpis*, are a potential *in vitro* model to understand the features of the infection-related adhesion phenomena [14]. These models are suitable for analyzing the effects of interactions between surface molecules from both parasite (e.g., gp63, LPG, PPG etc.) and host (e.g., proteoglycans) in the infection evolution.

Proteoglycans are characterized by a core protein that is covalently linked to glycosaminoglycan (GAG) side chains and are components of the extracellular matrix of insect [15,16] and mammalian tissues [17]. The GAGs structure shows linear polysaccharides constituted by repeating units of disaccharides containing uronic acid and a hexosamine; these disaccharides may vary in the type of hexosamine, hexose or hexuronic acid unit. The sulfated GAGs are classified as heparin [2-O-sulfo-β-D-glucuronic acid (GlcUA-2 S) or 2-O-sulfo-α-L-iduronic acid (IdoUA-2 S) associated to N-acetylglucosamine (GlcNAc) or N-sulfoglucosamine (GlcNS)], heparan sulfate [GlcUA, IdoUA or IdoUA-2 S associated to GlcNAc or GlcNS], chondroitin sulfate [GlcUA associated to N-acetylgalactosamine (GalNAc)], dermatan sulfate [GlcUA or IdoUA associated to GalNAc] and keratan sulfate [galactose (Gal) associated to GlcNAc], [18]. GAGs, as heparan sulfate and dermatan sulfate, present in host tissue have been reported to influence the *Leishmania* spp life cycle as well as of other parasites [19].

Although heparin is not found on the cell surface of the host, this GAG has been commonly used as a tool for studies on pathogen-host cell interactions. It has been previously shown that amastigotes of *L. (L.) amazonensis* and *Leishmania (Leishmania) major* have a greater ability to bind to heparin than promastigotes of these same species [20]. In addition, GAGs, including heparin, can induce the proliferation of *L. (L.) major* in the gut of the insect vector, increasing the parasite load of experimentally infected insects [21].

There is evidence that heparin-binding proteins (HBPs) present on the surface of *Leishmania* spp may play important roles in the parasites life cycle, defining the success of parasite attachment to and invasion of tissues of the mammalian and invertebrate hosts. In the parasite species in which these proteins have been identified, it was observed that HBPs present activity as adhesion proteins, and can promote the internalization and signaling in the host cells [22]. Experiments performed with promastigotes of *Leishmania (Leishmania) donovani* showed that about 860,000 units of these proteins are present on the surface of the parasite and that they are able to induce inhibition of protein kinase C activity in the host, through the binding to heparin [23,24]. Also, the expression of HBPs in *L. (L.) donovani* is related to the infective forms of this parasite: HBPs are predominant in stationary-phase promastigotes and successive culture passages of these parasites lead to a loss of the ability to bind to the heparin [25].

Previous reports from our group indicate that two HBPs (65.0 and 54.5 kDa) from *L. (V.) braziliensis* promastigotes recognize several molecules in the gut of *Lu. intermedia* and *Lu. whitmani*[26]. Both proteins are localized in the flagellar and membrane domain of the promastigotes but only the 65.0 kDa HBP presents metallo proteinase-like activity. Surface plasmon resonance analysis also demonstrated high-affinity binding between heparin and HBPs from the flagellar domain, forming a stable complex [27].

Focusing on fulfilling the lack of information about the interaction of *Leishmania* spp promastigotes and insect cell lines, as Lulo cells, we present the first evidence that HBPs from *L. (V.) braziliensis* promastigotes can be involved in parasite adhesion to these cells by a specific receptor.

## Methods

### Chemicals and reagents for culture

Schneider´s medium, Dulbecco's Modified Eagle Media (DMEM), detergents [Tween 20, and 3-[(3-cholamidopropyl)-dimethylammonium]- 1-propanesulfonate (CHAPS)], heparin sodium salt, biotinylated heparin, bovine serum albumin (BSA), 1,3-diaminepropane acetate, penicillin, streptomycin, Horseradish peroxidase (HRP) - conjugated goat anti-mouse IgG (anti-rabbit and anti-mouse HRP) were acquired from Sigma-Aldrich Chemical Co. (St. Louis, MO, USA). Heparin-Sepharose column (HiTrap-Heparin; 1.5 × 2.5 cm) was acquired from GE Healthcare. Fetal calf serum (FCS) was acquired from Cultilab S/A (Brazil). Electrophoresis reagents were acquired from BioRad Laboratories Inc. (US). Pre-Stained^TM^ Plus Protein Ladder was acquired from Fermentas Life Sciences (US). Amicon Centriprep YM-10 filter devices were acquired from Millipore (Billerica Inc, MA, USA). Anti-Na+/K + ATPase rabbit antibody was acquired from Abcam (Cambridge, MA, USA). Anti-glyceraldehyde-3-phosphate dehydrogenase (anti-GAPDH) mouse monoclonal antibody was acquired from Imgenex (San Diego, CA, USA). Chemiluminescence luminol reagent-ECL kit was acquired from Santa Cruz Biotechnology, Inc. (Santa Cruz, CA, USA). ^35^S-Na_2_SO_4_ was purchased from IPEN (São Paulo, SP, Brazil). Chondroitin 4-sulfate from whale cartilage was purchased from Seikagaku America, USA. Heparan sulfate from bovine pancreas was obtained from Opocrin Research Laboratories, Modena, Italy. Pronase and cetyltrimethylammonium bromide was from Merck, Darmstadt, Germany. All other reagents were of analytical grade or better.

### Parasites

Infective promastigotes of *L. (V.) braziliensis* (MCAN/BR/1998/619) were maintained at 28°C as a stock culture in Novy, MacNeal and Nicolle medium and subcultured every 4 days. Promastigote cultures were grown in Schneider´s medium supplemented with 10% heat-inactivated FCS until a density of 1 × 10^8^ cells/mL.

### Subcellular fractionation

Subcellular fractions enriched for flagella or surface membranes were obtained by centrifuging fractionation as previously described [27]. Promastigotes were washed twice by centrifugation (3,800 × g, 10 min, 4°C) in phosphate-buffered saline (pH 7.2), PBS, and then again washed twice in 10 mM Tris–HCl (pH 7.2) buffer containing 1 M NaCl, 0.2 M K_2_HPO_4_ and 0.5 M MgCl_2_. The cell pellet was then resuspended in 10 mM Tris–HCl (pH 7.5) containing 0.05 M sucrose (10 mL/g of cells) and disrupted in 1% CHAPS with 40 to 80 strokes in a Dounce-type homogenizer, following addition of sucrose to a final concentration of 0.25 M. Cells lysates were centrifuged (10 min, 4,300 × g, 4°C) and the supernatants were collected and centrifuged again (15 min, 12,000 × g, 4°C). The final pellet constituted the flagellar fraction (F_*f*_), whereas the supernatant was centrifuged again (45 min, 35,000 × g, 4°C) to obtain the pellet that constituted the membrane fraction (M_*f*_).

### Affinity chromatography

Heparin binding proteins were isolated from soluble proteins of F_f_ (HBP F_*f*_) or M_f_ (HBP M_*f*_) using a HiTrap-Heparin column previously equilibrated with 10 mM sodium phosphate pH 7.0. The bound proteins were eluted from the column using the equilibrium buffer containing of 2.0 M NaCl, as previously described [27]. The eluted fractions were concentrated using a Centriprep filter device, and the protein concentration was determined as previously described [28].

### Denaturant electrophoresis

Soluble proteins (40 μg) were resolved using 12% sodium dodecyl sulfate-polyacrylamide gel electrophoresis (SDS-PAGE) and silver staining, as previously described [29,30]. Electrophoresis was performed at 25°C in a Mini Protean II system (BioRad Laboratories, US).

### Western blot assays

For western blot assays, sample proteins (40 μg) were resolved using SDS-PAGE and, then, transferred onto 0.22 μm nitrocellulose membranes. Non-specific binding sites were blocked (4°C for 16 h) using a solution of 5% skimmed milk (w/v) in PBS with the addition of 0.5% Tween 20. The blots were washed six times with PBS containing 0.05% Tween 20 (PBST) and incubated (1 h, 25°C) with primary antibodies [anti-Na+/K + ATPase (1:500) or anti-GAPDH (1:100)], diluted in PBST. After six washes with PBST, the blots were incubated (1 h, 25°C) with anti-rabbit HRP (1:2500) or anti-mouse HRP (1:2000) diluted in PBST. The blots were once again washed as described above and the antibody binding was revealed by a chemiluminescence kit.

### Lulo cell line

The insect epithelioid cell line Lulo was cultured in a 1:1 mix of L15 (Leibovitz 1963) and Grace media supplemented with 10% FCS, penicillin (100U/mL) and streptomycin (100 μg/mL) at 28°C. The cells were seeded on coverlips to a final number of 2 x 10^5^ cells per well and grown to semiconfluent monolayers prior to interaction assays with parasites [14].

### Lulo cell metabolic labeling with radioactive sulfate

Lulo cells were maintained in culture medium free of sulfate for 24 h and then subjected to incorporation of ^35^ S-Na_2_SO_4_ (150 mCi/mL) in DMEM supplemented with 5% FBS and 10% horse serum (which have been dialyzed to remove the non-labeled sulfate). After 24 h incubation (28°C) with the labeled sulfate, the culture medium was removed and the cells were washed twice in PBS. In order to investigate the presence of GAGs at the cell surface, the cells were first detached from the dish with PBS containing 0.025% EDTA for 10 min. After centrifugation, the cell pellet was treated with 1 ml solution of 0.2% trypsin / 0.02% EDTA in PBS for 5 min (Cultilab, Campinas, SP) to remove the cell surface proteoglycans. Immediately, medium containing serum was added to neutralize trypsin. Following centrifugation, the supernatant was considered cell surface, and the pellet, cellular extract. The cell pellet was then lysed by treatment with 3.5 M urea in PBS. Proteoglycans from the three compartments (medium, cell surface and cell extracts) were precipitated with 3 vol. of ethanol (18 h, -20°C) in the presence of 200 μg of chondroitin 4-sulfate as a carrier. The pellets were dried and incubated (24 h, 37°C) with pronase (12 mg/mL) in 0.05 M Tris–HCl, pH 8.0. This GAG suspension was boiled (10 min, 100°C) and then dialyzed for 24 h with at least four changes of distilled water. Labeled GAGs were dried again and resuspended in 1 mL of water.

### GAGs and enzymatic analysis

5 μl of ^35^ S-GAGs´ samples were incubated with chondroitinases AC and ABC, and heparinase II (Sigma-Aldrich, St. Louis, MO, USA), according to the manufacturer´s instructions. The samples were applied to agarose gel slabs in 0.05 M 1,3-diaminepropane acetate buffer, pH 9.0 [31]. After electrophoresis, the gels were incubated (1 h, 25°C) with 0.2% cetyltrimethylammonium bromide, to induce GAGs precipitation. The gels were dried, and standard GAGs were stained with toluidine blue. To quantify the radiolabeled GAGs in the gel, a drop of known quantity of radioactive sulfate was applied and dried again. ^35^ S-radiolabeled GAGs, resistant to the enzymes, were visualized as bands, after exposure to a screen for five days, and identified using the image analysis system Cyclone (Storage Phosphor System–Packard Instrument).

### Lulo cells-*Leishmania* interaction

To evaluate the effect of HS or the HBPs fractions in the adherence of promastigotes to Lulo cells two different protocols were used: a) *L.(V.) braziliensis* promatigotes in a concentration of 4 x 10^7^ cells/mL, corresponding to 10:1 parasites/cells were pre-incubated (1 h, 4°C) with HS (20 μg/mL and 10 μg/mL), and then added to a semi confluent monolayer of Lulo cells; or, b) Lulo cells, in semi confluent monolayer, were incubated for 1 h with parasite proteins, (20 μg/ml HBP F_*f*_ or 10 μg/ml HBP M_*f*_) prior to the addition of parasites to the culture wells (4 x 10^7^ cells/mL). Subsequently, the Lulo cells/parasites cultures were kept in Grace/L-15 media for 2 h at 28°C. The chambers were extensively washed with PBS to remove non-adherent parasites, fixed with methanol and stained with Giemsa. The number of adherent promastigotes was determined by light microscopy examining 300 cells per well, and expressed as a relation of adhered parasites per 100 cells. Analysis was performed in triplicate.

### Binding assays by surface plasmon resonance (SPR)

SPR assays were performed using sensor chip with a carboxyl surface coated with neutravidin (Biocap; Nomadics, USA), as previously described [27]. Briefly, the chip surface was covered with biotinylated heparin (0.5 μg) and used in interaction with HBPs fractions (2 μg), BSA (0.1 - 0.001 μg) or whole promatigotes (1.4 × 10^5^ cells). To perform inhibition assays, promastigotes or HBPs fractions were pre-incubated (2 h, 4°C) with different concentrations of HS (0.1 μg - 0.001 μg). Prior to interaction with the sensor chips surface, the promastigotes were fixed (1 h, 4°C) with 1% paraformaldehyde and washed three times by centrifugation (800 × g, 10 min, 4°C) in PBS. SPR assays were performed at 25°C with 100 μL of material injected under a flow rate of 10 μL/min. The binding assays were performed in PBS and registered in real time using a sensorgram, where changes in the SPR angle (θspr) were measured as arbitrary resonance units (RU). Resonance signals of the samples were analyzed after subtraction of the RU values from the reference channel, to avoid methodology artifacts. SPR experiments were conducted in an optical biosensor SensiQ Pioneer (Icx Nomadics, USA) and the data were analyzed using Qdat software (Icx Nomadics, USA).

### Statistical analysis

To compare results Student’s test was applied, assuming equal variance between samples. Data matrices were considered statistically distinct when p-value was lower than 0.05.

## Results

**Figure 1:**
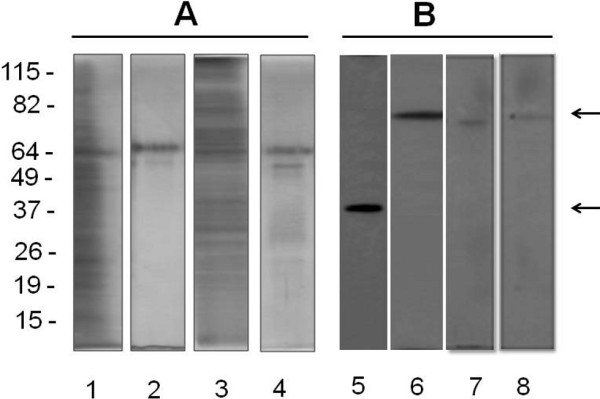
**Denaturant electrophoresis assays with*Leishmania (V.) braziliensis*proteins.** Protein samples (10 μg) of flagella (F_f_) and membrane (M_f_) fractions collected prior to heparin-sepharose column fractionation (1 and 3, respectively) or afterwards (HBP F_*f*_ - 2 and HBP M_*f*_ - 4) were applied into each slot, submitted to SDS-PAGE and revealed by silver staining (**A**). In parallel, protein samples (10 μg) from parasite cell lysates (5 and 6), F_*f*_ (7) and M_*f*_ (8) were separated by SDS-PAGE, transferred to a nitrocellulose membrane and revealed by Western blotting assays (**B**). In this last case, subcellular markers as Na+/K + ATPase (80 kDa) and GAPDH (36 kDa) proteins (arrows) were detected using specific antibodies and an HRP-conjugated secondary antibody. Bands were visualized by chemiluminescence. These results are representative of four independent experiments.

Two subcellular fractions from *L. (V.) brasiliensis* were characterized in this manuscript. The protein profiles of flagellar (F_f_) and membrane (M_f_) fractions, as well as their corresponding heparin-binding subfractions (HBP F_f_ and HBP M_f_), were analyzed by SDS-PAGE revealing complex features, with proteins varying from 17 kDa to 115 kDa. The analysis of isolated proteins from HBP F_f_ and HBP M_f_, using affinity column, showed two main protein bands, 65.0 and 55.0 kDa, for both fractions, as revealed by silver staining (Figure 1A).

The effectiveness of the employed subcellular fractionation technique to separate flagella (F_*f*_) and membrane (M_*f*_) fractions was confirmed during our study. Western blot analyses (Figure 1B) indicated that the obtained F_*f*_ and M_*f*_ fractions were enriched with Na+/K + ATPase (80 kDa protein band), which is a typical protein of membrane and flagella. Additionally, the levels of GAPDH protein (36 kDa), a known cytosolic contaminant were undetectable in the samples, thus indicating their purity. Both proteins were detected in the extract from whole parasite, showing that these proteins can be found in the parasite (Figure 1B).

Characterization of F_*f*_ and M_*f*_ was performed by detecting typical organelles of these cellular components, as flagellum fragments or spherical membrane-bound vesicles, respectively, using transmission electron microscopy, as previously described (data not shown) [27]

**Figure 2:**
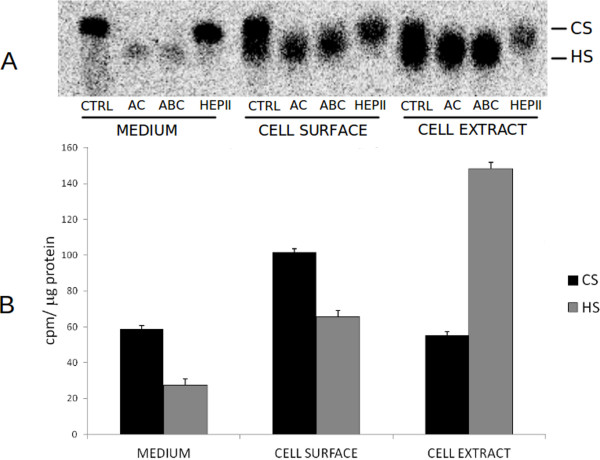
**Quantification and identification of sulfated glycosaminoglycans in Lulo cells.** After metabolic labeling of Lulo cells with ^35^ S-Na_2_SO_4_, cell extract, cell surface and cell culture medium were subjected to enzymatic degradation and afterwards to gel electrophoresis in 0.6% agarose. The bands were identified using an image analysis system, the Cyclone® Storage Phosphor System-Packard Instrument (**A**). Quantification was performed by densitometry with Opti-Quanti Software and expressed as cpm/mg protein (**B**). Defined quantities of non-labeled GAGs (chondroitin sulfate - CS; heparan sulfate - HS) were used as standard control; CTRL – incubation control with buffer; AC – chondroitinase AC; ABC – chondroitinase ABC; HEP II – heparinase II.

To analyze whether GAGs were expressed by Lulo cells *in vitro* and, thus, would be able to influence parasite adhesion, experiments for the detection of radioactive labeled GAGs were carried out. We investigated GAGs from cell-secreted material in the growth medium, cell extracts and most importantly, cell surface. The GAGs were metabolically labeled by adding radioactive sulfate ([^35^ S]-Na_2_SO_4_) to cell growth medium and, afterwards purified samples from these compartments were analyzed by agarose gel electrophoresis for the presence of radioactivity. After 24 h in culture, Lulo cells expressed both heparan sulfate and chondroitin sulfate (Figure 2 A and B). The presence of these molecules on cell surfaces was further confirmed by treating GAGs from each compartment with heparinase II and chondroitinase AC and ABC (Figure 2 A).

**Figure 3:**
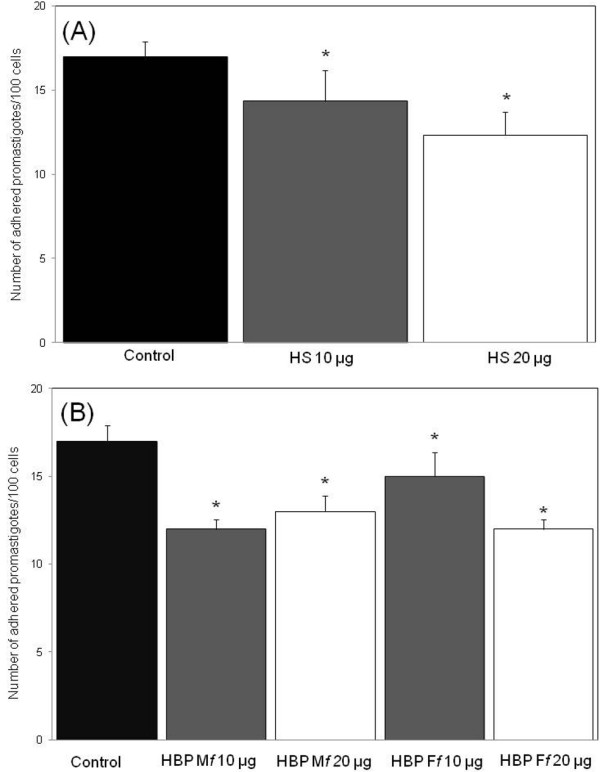
**Quantification of promastigote adhesion to Lulo cells.** Adhesion inhibition assays were performed using promastigotes without or with pre-incubation with increasing concentrations of heparan sulfate (**A**) were co-incubated (28°C) with Lulo cells monolayers. Likewise, Lulo cells monolayers without or with pre-incubation with different concentrations (10 μg and 20 μg) of HBP F_*f*_ and HBP M_*f*,_ and 20 μg of BSA (control) were co-incubated with promastigotes (**B**). The coverslip containing cells and/or parasites samples were stained with Giemsa and the number of adhered promastigotes/100 cells was determined by light microscopy examining 300 cells per coverslip, in triplicate. Data are expressed as percentile values (%) and represent average and standard deviation of five independent experiments - (*), p < 0.05.

Further, we addressed the question whether heparan sulfate (HS) interaction with the parasites may be involved in their adhesion to host cell (Figure 3). To assess the role of HS in the adhesion of promastigotes to Lulo cells, a competition assay was performed, where promastigotes were treated with HS prior to interaction with Lulo cells. In the control assays performed with Lulo cell monolayers pre-incubated or not with BSA (20 μg), the percentage of adhered parasites was 17 ± (2.0). The quantitative data revealed a significant decrease in the number of adhered promastigotes/100 cells: a reduction of 30% (for HS 20 μg/ml; p = 0.0025) and 16% (for HS10μg/ml; p = 0.0025), when compared to the control (Figure 3 A). Also, pre-incubation of Lulo cell monolayers with HBP_f_ or HBP_M_ was able to induce a reduction in the adhesion rates, when compared to the control: 35.2% (for HBP M_f_ 10 μg; p = 0.0018), 25.4% (for HBP M_f_ 20 μg; p = 0.0028), 10.0% (for HBP F_f_ 10 μg; p = 0.066) and 31.4% (for HBP F_f_ 20 μg; p = 0.0071), (Figure 3 B).

**Figure 4:**
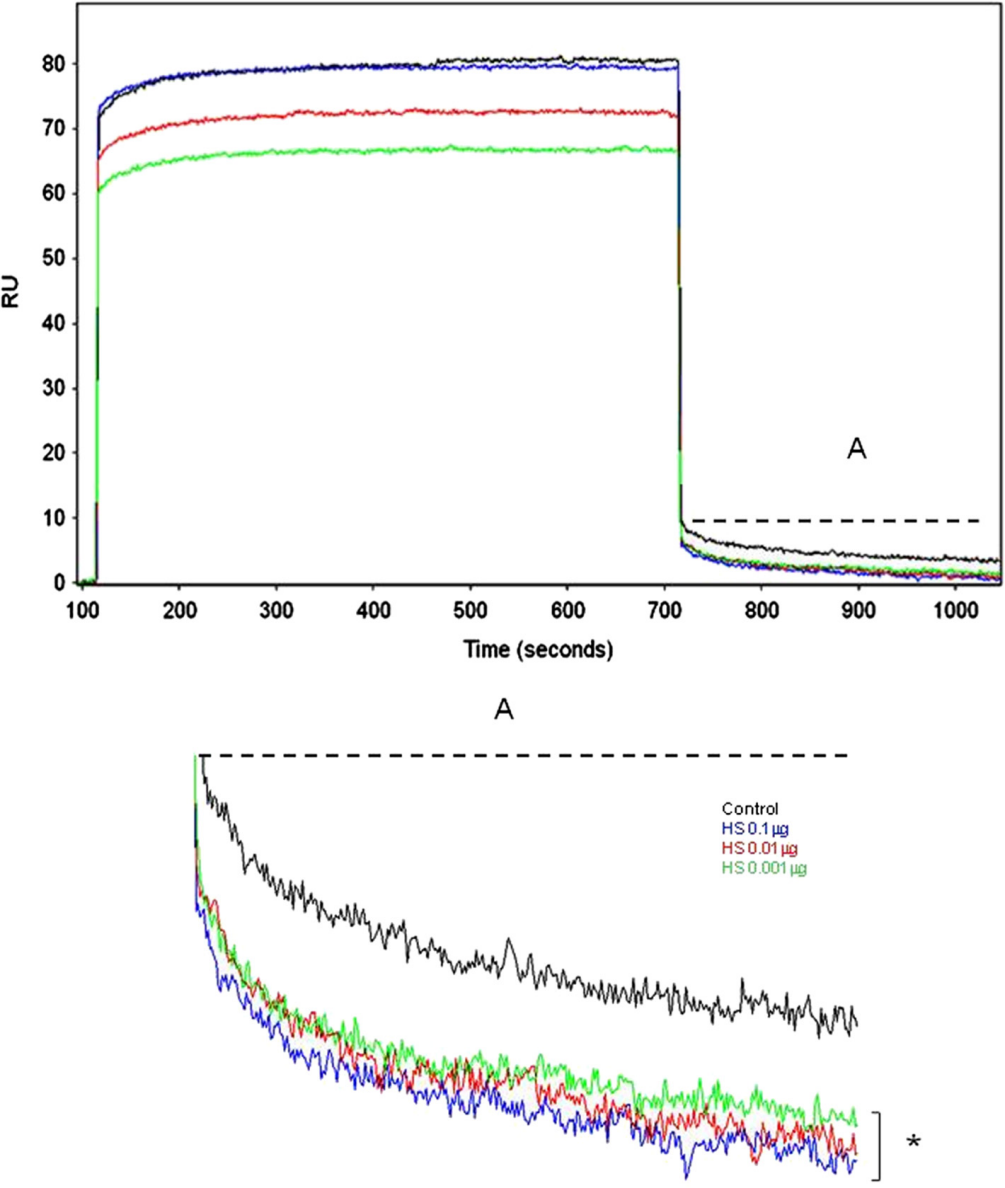
**Analysis of*Leishmania. (V.) braziliensis*promastigotes adhesion to glycosaminoglycans by surface plasmon resonance.** Sensor chips were covered with biotinylated heparin, and promastigotes were passed over the chip surface. The parasites were assayed without pre-incubation with heparan sulfate (black line) or after pre-incubation with 0.001 μg (green line), 0.01 μg (red line) or 0.1 μg (blue line) of heparan sulfate. Significant decrease in adhesion rates was observed in the pre-incubated samples was achieved - (*), p < 0.05. Data are presented as arbitrary resonance units (RU) and are representative of two independent experiments**.** The dissociation RU values are representative of the average response between 720 and 1020 seconds (A) in all assays.

Complementary to the data obtained from the radioactive labeling assays, it was also possible to assess the presence of GAGs ligands on the surface of *L. (V.) braziliensis* promastigotes through the SPR assays. The binding sensorgram graphs, after the injection of parasites onto a chip surface functionalized with heparin indicated resonance units (RU) values of 80 and 12, respectively (Figure 4). The specificity of this binding was confirmed by additional SPR assays, in which promastigotes were incubated with increasing concentrations of HS prior to injection on the chip. In these assays, it was possible to observe a significant inhibition (p = 0.001) of promastigotes binding to immobilized heparin, with RU values decreasing by 38% when parasites were incubated with 0.1, 0.01 and 0.001 μg/ml of HS (Figure 4).

The binding potential of HBP F_*f*_ and HBP M_*f*_ to immobilized heparin was also assessed. The results show that the binding of these fractions to immobilized heparin (approximately 70 RU) is inhibited by incubation with HS (0.1, 0.01 and 0.001 μg/ml) leading to a decrease in RU values to 32 ± 7.2 (for HBP F_*f*_) and 48 ± 17 (HBP M_*f*_ ) for all assayed concentration (data not shown).

The specificity of the binding in the SPR assays was confirmed by negative controls using immobilized BSA on the chip surface. In these control experiments, no significant association and dissociation rates were detected for tests with promastigotes or either protein fractions, as previously described [27].

## Discussion

The *Leishmania* spp parasites are an important example of protozoans related to human infectious diseases with transmission by arthropods [32,33]. Some current studies on the life cycle of these parasites have emphasized the interaction of promastigotes with gut cells or tissues of sandflies [34,35]. In this context, it has been shown recently that the Lulo cell line can be useful as a model for studies of insect-parasite interactions for *Leishmania* spp [14], partially simulating the adhesion events that occur in the sandflies gut. In the present study, we show evidence that 65.0 kDa and 55.0 kDa HBPs from the promastigote surface can participate in the adhesion to Lulo cells through HS binding.

The Lulo cell line is composed of epitheloid cells, originated from embryonic tissue of *Lu. longipalpis* adult insects, a species which is commonly used in studies of interaction between parasite and insect [36]. Although Lulo cells have already been described as interacting more avidly with *L. (L.) chagasi* promastigotes of [14,37], we provide, in the present study, additional evidence that *L. (V.) braziliensis* has the ability to interact with Lulo cells. This is an interesting finding, as *Lu. longipalpis* has been reported as an efficient vector for species of the subgenera *Leishmania* and Lulo cells are derived from this sand fly species. Possibly, these data are indicative of the existence of common molecules between the subgenera *Viannia* and *Leishmania*, which would be related to promastigote adhesion to the surface of Lulo cells.

Since it is known that glycosaminoglycan-binding microbial proteins interfere in the processes of adhesion and invasion of host tissues [38] and that these proteins have already been described on the surface of *L. (V.) braziliensis*, we considered it would be interesting to evaluate the participation of these proteins in the attachment of promastigotes to Lulo cells. The data gathered in the present study, corroborates the influence of HBPs in the promastigote adhesion to Lulo cells, supporting the involvement of parasite surface compounds in the adhesion to host cells [4,7]. Although we have previously described that HBPs from flagella have more affinity to bind to heparin [27], the data presented here prove that HBPs from both flagella and membrane fractions are able to participate in the adhesion of promatigotes to the Lulo cell monolayers, interacting with heparan sulfate molecules of these cells. These findings are supported by previous studies that indicate the participation of sulfated glycosaminoglycans in host-parasite recognition processes, as: invasion of hepatocytes by *Plasmodium* sporozoites through the major surface proteins CSP and TRAP [39,40]; adhesion of *Plasmodium* sp.-infected erythrocytes to the placenta [41]; invasion of mosquito midgut by *Plasmodium falciparum*[42]; and, adhesion of *Trypanosoma* epimastigotes to the gut epithelial cells of *Rhodnius prolixus*[43,44].

Additionally, this study presents the first report of the presence of sulfated glycosaminoglycans, HS and CS, in Lulo cells. Although the present results do not directly specify the localization of these components on the cell surface, the competition assays are indicative of a surface localization of glycosaminoglycans on the Lulo cells. Indeed, the current literature in biochemistry shows that sulfated glycosaminoglycans are ubiquitous among animal cell membranes and are present on the surface of virtually every adherent cell [45] and can modulate the actions of a large number of extracellular ligands [46]. Generally, invertebrates produce the same types of GAGs as vertebrates, except that hyaluronic acid is not present and chondroitin chains tend to be non-sulfated [18]. Thus, our results add new information to the description of the diversity of GAGs distributed in cells and tissues of invertebrates.

Due to the stability of the HBP-GAG complexes observed in the biosensor analysis, we postulate that HBPs play specific roles in parasite-host interactions. It is the second time we are able to demonstrate the potential of SPR biosensor to prove the surface localization of HBPs on promastigotes. The reduction of the RU values observed in the competition assays with promastigotes or HS reinforced the conclusion that the HBPs can recognize the actual GAGs in the Lulo cells surface, which is in agreement with the Lulo cell-promastigotes interaction data. Moreover, the slow decrease in the sensorgram baseline after interaction of HBP fractions with the heparin-coated chip surface is evidence of the stability of the complex formed between promastigote HBPs and GAGs, as HS.

Biosensing surface procedures have already been used to detect specific proteins on the surface of *Trypanosoma cruzi* epimastigotes [44] and *L. (V.) braziliensis* promastigotes [27] and correlate to stable interactions that occur at the interface of these protozoan parasites to their host cells surface. Similar analysis has also been conducted to study the interaction of measles virus and heparin in the infection of SLAM-negative cell lines [47] and of *Plasmodium* circumsporozoite with heparin in liver invasion [48].

The strong binding of *L. (V.) braziliensis* to GAGs observed in the performed physicochemical assays reinforces the putative function of the 65.0 kDa and 55.0 kDa proteins of promastigotes as important adherent compounds in the life cycle of this parasite. In prior studies, proteins with similar molecular masses were isolated by heparin-affinity chromatography methodology and described as hydrophobic proteins of *L. (V.) braziliensis* promastigotes [26]. As we have previously described a 65.0 kDa HBP of *L. (V.) braziliensis* with metallo proteinase-like properties [27], we hypothesize that this HBP may act as a protagonist of proteolytic activities triggered by the HBPs-GAGs recognition event and thus play a role for the adhesion of the parasite to insect cells. Additionally, it has also been suggested that *Leishmania* spp parasites could modulate key enzymes or proteins in the gut of the sandfly, therefore obtaining advantages for their establishment and survival in these hosts [49]. Collectively, our data suggest that metallo proteinase from promastigote surfaces are involved in the attachment to Lulo cells via binding of GAGs, as HS. Additional studies will be necessary to further prove this hypothesis and also investigate the biological role of the 55 kDa HBP.

## Conclusions

We have presented evidence that HBPs (with 65.0 kDa and 55.0 kDa) from the membrane and flagella of *L. (V.) braziliensis* promastigotes have the ability to recognize HS, with stable receptor-ligand interaction. The set of results presented here emphasizes the role of HBPs on promastigote adhesion to the Lulo cells by their GAGs.

## Competing interests

The authors declare that they have no competing interests.

## Authors’ contributions

LMCC, MCSP and CRA formulated the idea and wrote the manuscript; LMCC, FSS, FOOJ, ROAS, CMV, and MFM performed the experimental assays. BASP, LT, HBN, RPB and FB provided critical comments and participated in protocol drafting, results analysis and discussion construction. All authors approved the final version of this manuscript
